# A Herbal Mixture of Sesami Semen Nigrum and Longan Arillus Induces Neurite Outgrowth in Cultured Neurons and Shows Anti-Depression in Chronic Mild Stress-Induced Rats

**DOI:** 10.1155/2022/8809546

**Published:** 2022-06-16

**Authors:** Alex Xiong Gao, Tracy Chen-Xi Xia, Marvin Shing-Hung Mak, Kevin Yue Zhu, Tina Ting-Xia Dong, Karl Wah-Keung Tsim

**Affiliations:** ^1^Shenzhen Key Laboratory of Edible and Medicinal Bioresources, HKUST Shenzhen Research Institute, Hi-Tech Park, Nanshan, Shenzhen 518000, China; ^2^Division of Life Science and Center for Chinese Medicine, The Hong Kong University of Science and Technology, Clear Water Bay, Hong Kong, China; ^3^School of Pharmacy, Nanjing University of Chinese Medicine, Nanjing, 210023 Jiangsu, China

## Abstract

Medicinal food homology is referring to a group of food itself being considered as herbal medicine without a boundary of usage. Under the guidance of this food/medicine principle, the current study aims to develop anti-depressant from this food/medicine catalog. The herbal mixture of Sesami Semen Nigrum and Longan Arillus was evaluated in cultured PC12 rat pheochromocytoma cells, rat primary cortical neurons, and in chronic mild stress (CMS)-induced depressive rat model. The combination of two ethanolic extracts of Sesami Semen Nigrum and Longan Arillus in 1 : 1 ratio mimicked the function of nerve growth factor (NGF) and synergistically induced neurite outgrowth of PC12 cells. Besides, the expression and phosphorylation of tropomyosin receptor kinase A (TrkA) of the cultured cells were also elevated. This neurotrophic activity of herbal mixture was further supported by the increased expressions of biomarkers for neurogenesis and synaptogenesis in cortical neurons. Moreover, the depressed rats were soothed by the intake of herbal mixture, showing improved performance in behavior tests, as well as reversed levels of neurotransmitters and neurotrophic factors. Our results provide a new way to make full use of the current food/medicine resources, as to accelerate the development of therapeutics for depression.

## 1. Introduction

Depression, or major depressive disorder, is a widely occurring mental disease that severely affects people's daily lives, even resulting in suicide tendencies [1]. As announced by the World Health Organization (WHO) in 2020, there are more than 264 million patients having depression at all ages worldwide. Each year, around 0.8 million suicide cases are reported. Depression can be developed both physiologically and mentally, thus psychosocial treatments and anti-depressants are two major therapies for this global disorder. Behavioral activation, cognitive therapy, and interpersonal psychotherapy are common psychosocial treatments, while selective serotonin reuptake inhibitors and tricyclic anti-depressants are common anti-depressants that have been shown to have fast action and effectiveness in treatment [2]. However, the side effects of synthetic anti-depressants are frequently reported, including cardiotoxicity, hypertensive crisis, sexual dysfunction, and sleep disorder [3].

To search alternative medical treatment, Chinese herbal medicines are popular alternatives in treating depression because of their less harmful adverse effects [4]. Having the support from the natural therapy, there is always a misconception of thinking that herbal medicines are “toxic-free.” These misunderstandings are not only a result of the ceaseless advertisements but also due to lack of systematic pharmacological reports on the elevating toxicity of Chinese herbal medicine. Indeed, a list of 245 species of Chinese medicine (out of total 365 species being recorded) was recorded as potentially toxic, or even poisonous, in Shennong Bencao Jing < Shennong's Classic of Materia Medica > (∼200 B.C.). Another limitation of the global popularization of herbal medicine is the limited resources and stringent growing environment, i.e., the specific requirement of growth environment for different herbs. The limited supply of herbal medicines could result in abnormal prices and illegal harvesting and trading.

Among those herbal medicines, there is a group of medicines that are considered as common food, and which is considered as medicinal food homology. This group of medicine/food has not been fully utilized. To find drug targets for anti-depression, we screened the herbal candidates under the list of medicinal food homology that could induce the differentiation of cultured PC12 cells [5, 6]. Among the positive hits, two major “food” ingredients, Sesami Semen Nigrum (the dried ripe seed of *Sesamum indicum* L.) and Longan Arillus (the aril of *Dimocarpus longan* Lour.) were identified and chosen for further evaluation. According to Chinese medicine theory, both herbs are known to have tonic effects. Longan Arillus is commonly consumed as functional food to improve insomnia, shock, and forgetfulness, and Sesami Semen Nigrum is often prescribed for the treatment of dizziness, tinnitus, impaired hearing, and constipation.

The chronic mild stress (CMS)-induced depressive rat model is one of the most effective and widely used animal models of depression [7, 8]. By continuously exerting stimuli, e.g., physical stress and external material environment stress, with reduced intensity, the CMS model realistically simulates the “difficulties” being encountered in people's daily lives. Meanwhile, the order of applied stress factors is randomly arranged in the experiment so that the animals cannot predict the occurrence of challenges. This chronic and unpredictable stress on the animals results in body dysfunction, e.g., a decrease in physical exercise ability and increased plasma corticosteroids, similar to the depressive symptoms of humans. Chronic stress-mediated behavioral abnormalities can be maintained for several months, and the usage of anti-depressants can correct these abnormal behaviors. Therefore, it is an ideal and reliable animal model of depression. Besides, the consumption of sucrose water as well as the mobility of forced swimming testing could reflect the loss of pleasure or the degree of depression [7]. Here, the herbal mixture of Sesami Semen Nigrum and Longan Arillus at 1 : 1 ratio was revealed to induce neuronal differentiation in neuronal cells and to restore, significantly, the CMS-induced depressive rats. Moreover, the signaling being triggered by the herbal mixtures was revealed.

## 2. Materials and Methods

### 2.1. Chemicals and Herbal Materials

Sesamin and adenosine, >98% purity as determined by HPLC, were purchased from Yuanye Biotechnology (Shanghai, China). Fluoxetine, dopamine, noradrenaline, and 5-hydroxytryptamine were obtained from Sigma-Aldrich (St. Louis, MO). Sesami Semen Nigrum (the dried ripe seed of *S. indicum* of the family Pedaliaceae) and Longan Arillus (the dried aril of *D. longan* of the family Sapindaceae) were obtained from commercial sources. Dr. X. Y. Yang from the Yunnan Institute of Materia Medica conducted morphology authentication following the Chinese Pharmacopoeia (2020 edition). Voucher specimens of the herbs were stored in our laboratory. 50 g of herbal powder was extracted twice by sonication in 1 L of 50% ethanol for 30 min [6]. After concentration and lyophilization, the dried extracts were collected. The yield of extract was 2.06% (w/w) and 60.11% (w/w) for SSN and LA, respectively.

### 2.2. HPLC Analysis

The extracts of Sesami Semen Nigrum and Longan Arillus were analyzed by HPLC-DAD (Agilent 1200) with a C_18_ column (4.6 × 250 mm, 5 *μ*m) at an absorbance of 285 and 260 nm, respectively. The mobile phases were (A) 0.2% diluted aqueous formic acid and (B) acetonitrile. The gradient program indicated by mobile phase B was described as follows: 0–5 min, isocratic gradient 5%; 5–20 min, linear gradient 5–10%; 20–40 min, linear gradient 10–25%; 40–55 min, linear gradient 25–35%; 55–65 min, linear gradient 35–40%; 65–90 min, linear gradient 40–100%; and 90–96 min, isocratic gradient 100%. The injection volume was set as 10 *μ*L, and the flow rate was 1.0 mL/min.

### 2.3. Cell Culture

PC12 cells purchased from the American Type Culture Collection were cultured in a humidified incubator (37°C, 5% CO_2_) DMEM (6% FBS + 6% HS) was used for cell subculture, as described before [9]. Primary rat cortical neurons were isolated from Sprague-Dawley (SD) rat embryos at Day 18, as described in previous studies [10]. Neurobasal medium (added with B-27 supplement, Thermo Fisher Scientific, Waltham, MA) was used to maintain the cultures. For the detection of neuritogenesis and synaptogenesis in cortical neurons, the cultures were treated at 3 DIV with the herbal mixture (0.1–10 *μ*g/mL) for another 3 days. Cultured neurons were stained with antibodies against MAP2 and synapsin-1, followed by fluorescent secondary antibodies (Cell Signaling Technology, Danvers, MA). The levels of MAP2 and synapsin-1 were observed under the fluorescent microscope. The averaged axonal length and the number of synapsin 1 puncta (per 100 *μ*m) were calculated according to 30 randomly selected neurons.

### 2.4. Neurite Outgrowth of PC12 Cells

PC12 cells cultured in DMEM (1% FBS + 1% HS) were treated with herbal extracts or NGF for 72 h, and fresh medium with the drug was changed every 24 h. Morphology images were captured under a phase-contrast light microscope. Cells with any neurites longer than the cell body diameter were regarded as differentiated. For each culture, at least 5 random visual fields, with approximately 100 cells, were counted for the quantification.

### 2.5. SDS-PAGE and Western Blotting

Cell lysates were collected, and protein samples (∼40 *μ*g) were separated on 8% SDS-PAGE gels. Nitrocellulose membranes were used for transferring the separated proteins, which afterward were blocked by 2.5% BSA with TBST (TBS + 0.1% Tween 20) and incubated with primary antibody diluted in 2.5% BSA in TBST overnight at 4°C. Primary antibodies, phospho-tropomyosin receptor kinase A (TrkA), TrkA and *β*-actin, were purchased from Cell Signaling Technology. The membrane was washed and then incubated at room temperature for 1 h with an HRP-conjugated secondary antibody (Cell Signaling Technology). The immune complexes were photographed under the ChemiDoc system (Bio-Rad Laboratories Inc, Hercules, CA.) through the enhanced chemiluminescence (ECL) method (Thermo Fisher Scientific). The protein content was determined by Braford reagent (Bio-Rad).

### 2.6. Animal Experiments

Sprague–Dawley (SD) rats (male, 150–180 g, 7 weeks old) were purchased from Shanghai SIPPR BK Laboratory Animals Ltd. (Shanghai, China). Animals were supplied with standard diet and water without restriction and maintained under the following environment: 12 h light/dark cycle (6 : 00 a.m. to 6 : 00 p.m., lights on; 6 : 00 p.m. to 6 : 00 a.m. of next day, light off); temperature set as 22 ± 2°C; and humidity set as 50 ± 10%. The animal experimental protocol (No. 2015–0254) was under the approval of the Animal Ethics Committee of China Pharmaceutical University and guided by Principles of Laboratory Animal Care (NIH publication No. 80–23, revised 1996). The experimental environment was kept quiet with soft light when establishing the chronic mild stress (CMS)-induced rat depressive model. Animals in the control group were not disturbed unless under house maintenance, e.g., cage cleaning. The CMS procedures adopted from previous studies with adjustments were described as follows: (1) 24 h of deprived water supply, (2) 10 h under stroboscopic illumination, (3) 7 h in 45° tilted cage, (4) 10 h in a noisy environment, (5) 12 h in the cage soiled by 200 mL water in 100 g sawdust bedding, (6) 1 h of exposure to an empty bottle, (7) 6 min of forced swimming at 8°C, (8) 6 min of tail-clipping, and (9) 21 h of deprived food supply [7, 11]. The experiment lasted for 6 weeks with the above 9 stressors, randomly arranged every one week. After the CMS cycles, the sucrose test and forced swimming test were performed for the evaluation of depressive rats.

For the sucrose preference test, animals in all groups were taught to be adapted with two bottles of 1% sucrose solution (w/v) for 24 h. After that, one bottle was replaced with tap water and lasted for another 24 h. Then, animals were arranged in water and food deprivation for 24 h. When sucrose preference test started, animals were kept in individual cages with two bottles provided (100 mL 1% sucrose solution (w/v) and 100 mL water, respectively). After 3 h, the consumed volumes of sucrose solution and water were recorded. Sucrose preference = (sucrose consumption/(water consumption + sucrose consumption)) × 100%. For the forced swimming test, the animals were sent for a 50 cm (height) × 20 cm (diameter) glass cylinder filled with 30 cm height of water at 22 ± 2°C. Animals were forced into preswimming for 15 min. 24 h later, the swimming behavior, i.e., the latency to float was recorded for 5 min [12].

### 2.7. Measurement of Neurotransmitters

Manipulated rats were sacrificed by decapitation. Whole brain tissues were separated and rapidly frozen in liquid nitrogen. Every 1 g sample was treated with 5 mL lysate (0.6 M perchloric acid, 0.5 mM Na_2_EDTA, 0.1 g/L L-cysteine) and centrifuged (14,000*g* × 15 min, 4°C) twice. The supernatant was added with perchloric acid precipitation agent (0.6 mol/L perchloric acid, 1.2 MK_2_HPO_4_, and 2 mM Na_2_EDTA), followed by centrifugation (14,000*g* × 15 min, 4°C) and filtration. Chromatographic separation of neurotransmitters was carried out by using the Shimadzu HPLC-RF series equipped with an Agilent ZORBAX SB-C_18_ column (150 mm × 4.6 mm, 5 *μ*m). The mobile phases were: (A) citric acid-sodium acetate buffer (50 mM citric acid, 50 mM sodium acetate, 0.5 mM 1-heptane sulfonate, 5 mM triethylamine, and 0.5 mM Na_2_EDTA) and (B) methanol (95 : 5, v/v, pH 3.8). The flow rate was set as 1.0 mL/min; the injection volume was 5 *μ*L; emission wavelength was 330 nm; and the excitation wavelength was 280 nm [13].

### 2.8. Real-Time Quantitative PCR

RNA was extracted with RNAzol reagent, followed by reverse transcription via M-MLV reverse transcriptase. The cDNA was sent for real-time PCR analysis by using the Mx3000p™ multiplex quantitative PCR machine (BD Biosciences Clontech, San Jose, CA). Primers were listed in Table S1.

### 2.9. Statistical Analysis

All data were analyzed using one-way ANOVA followed by the Bonferroni post hoc test. Statistical significance was determined as (*∗*) when *p* < 0.05.

## 3. Results

### 3.1. The Herbal Mixture Induces Neurite Outgrowth

Promoting neurogenesis and synaptogenesis stands for a novel direction in treating depression [8, 14]. In HerboChip® drug screening platform, the biotinylated-NGF was used to screen the chip being coated with HPLC-separated fractions from herbal extracts [5, 6]. Eleven herbal extracts, deriving from a medicine/food catalog, with obvious binding signals with NGF were identified. In rat pheochromocytoma PC12 cells, a classic cell model in evaluating the neurogenetic activity of drug candidates [15, 16], these herbal extracts induced cell differentiation. Among these extracts, Sesami Semen Nigrum (the dried ripe seed of *S. indicum*) and Longan Arillus (the aril of *D. longan*), as well as their combination, showed the optimal ability to promote neuronal differentiation and therefore which were chosen for further exploration.

**Figure 1:**
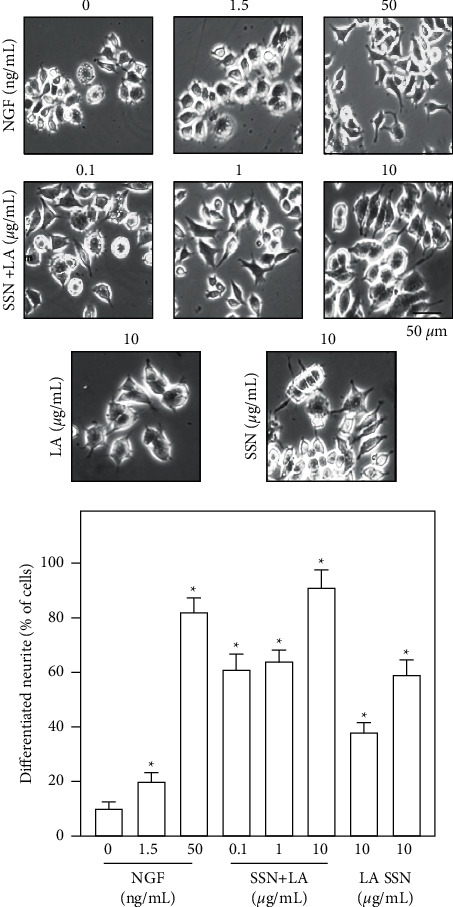
Herbal mixture of Sesami Semen Nigrum and Longan Arillus induces neuronal differentiation. PC12 cells were seeded onto a 6-well plate and cultured for 24 h, then the medium was replaced with low DMEM with 1% FBS and 1% HS in the presence of Sesami Semen Nigrum (SSN) and/or Longan Arillus (LA) or NGF. Fresh medium with drug was changed every 24 h. Images were taken after 3 days of treatment, and representative pictures are shown (upper panel). The percentage of differentiated cells was calculated and plotted (lower panel). Values are expressed in percentage of total number of cells, in mean ± SEM, *n* = 4. ( ^*∗*^) *p* < 0.05.

**Figure 2:**
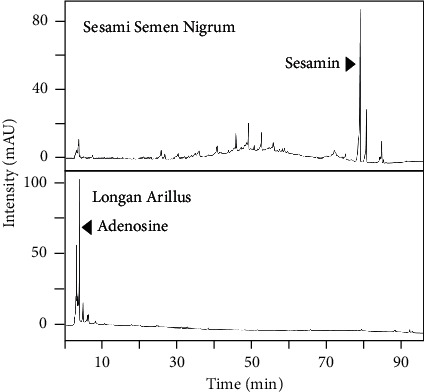
HPLC fingerprints of extracts of Sesami Semen Nigrum and Longan Arillus. The ethanolic extracts of Sesami Semen Nigrum (10 mg/mL) and Longan Arillus (100 mg/mL) were subjected to HPLC chromatograms with a DAD detector at an absorbance of 285 nm and 260 nm, respectively. The peaks corresponding to adenosine and sesamin are indicated. A representative fingerprint is shown, *n* = 3.

In cultured PC12 cells, application of NGF induced the growth of neurites, as well as the number of cells having extended neurites, in a dose-dependent manner, and therefore 50 ng/mL NGF was served as a positive control (Figure 1). The ethanolic extract of Sesami Semen Nigrum, or Longan Arillus, did not show cell toxicity up to 1 mg/mL of extract (data not shown). Both herbal extracts induced neurite outgrowth similar to that of NGF. In the synergistic analysis, the combination of two herbal extracts, Sesami Semen Nigrum and Longan Arillus, at a weight ratio of 1 : 1, synergistically induced the cell differentiation (Figure 1). Other ratios were tested but not as good as that of the 1 : 1 ratio. The maximal induction of cell differentiation was at 10 *μ*g/mL of herbal mixture, i.e., 5 *μ*g/mL Sesami Semen Nigrum + 5 *μ*g/mL Longan Arillus, which was more efficient than the positive control NGF at 50 ng/mL, stimulating nearly 90% of cells to differentiate. The herbal extract was then standardized by HPLC chromatography with respective compounds for Sesami Semen Nigrum and Longan Arillus, i.e., sesamin and adenosine (Figure 2). This chemical fingerprint could serve as parameters to repeat the consequent experiments.

**Figure 3:**
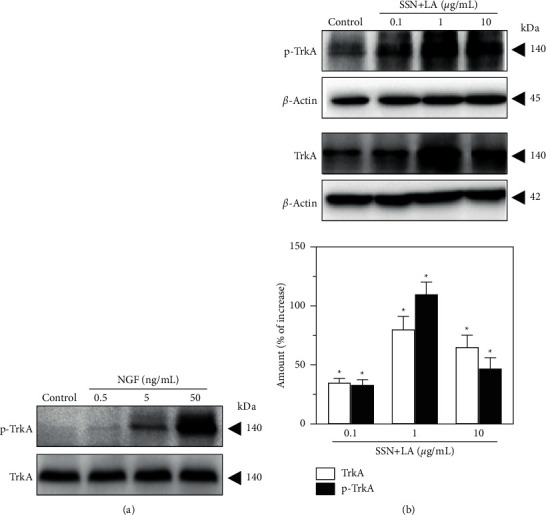
Combined extracts of Sesami Semen Nigrum and Longan Arillus upregulate TrkA signaling. (a) Serum-starved PC12 cells were treated with NGF (0–50 ng/mL) for 15 min. The protein levels of total-TrkA (∼140 kDa) and phosphorylated TrkA (∼140 kDa) were determined. Representative blots are shown. (b) The herbal extracts of Sesami Semen Nigrum (SSN) and Longan Arillus (LA) in 1 : 1 ratio, i.e., 10 *μ*g/mL SSN + LA was referring to 5 *μ*g/mL each, was employed. After 48 h of herbal treatment in low DMEM, PC12 cells were harvested and protein levels of total-TrkA and phosphorylated TrkA were determined by Western blot. Representative blots are shown (upper panel), and quantification results are plotted (lower panel). *β*-Actin (∼45 kDa) was used as a loading control. Values are expressed in percentage of increase against the untreated control, in mean ± SEM, *n* = 4. (*∗*) *p* < 0.05.

NGF stimulates the downstream signaling pathways through its specific receptor TrkA, leading to neuronal differentiation and protecting the cells from being attacked by various toxins [17]. As expected in cultured PC12 cells, the applied NGF-induced Trk phosphorylation in a dose-dependent manner (Figure 3(a)). Because of the herbal mixture's having an NGF-like effect, the levels of expression and phosphorylation of TrkA were tested here. After 48 hours of the treatment of mixed herbal extracts in the cultures, total TrkA and phosphorylated TrkA (both at ∼140 kDa) were increased, markedly, to ∼2 folds under 1 *μ*g/mL of the mixture (Figure 3(b)). The results implied that the cells upon incubation with the herbal mixture could strengthen TrkA signaling as well as possibly increase the cell sensitivity to NGF.

**Figure 4:**
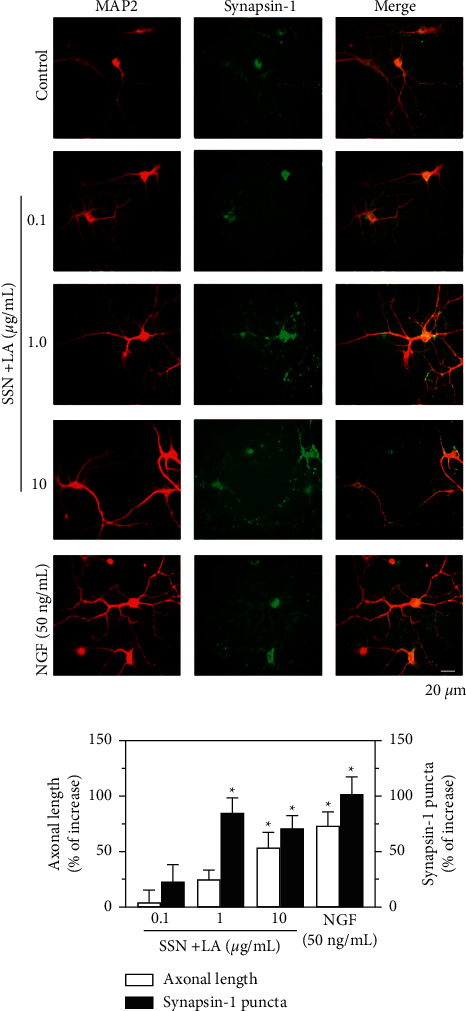
Combined extracts of Sesami Semen Nigrum and Longan Arillus promote neurogenesis and synaptogenesis. Cultured rat cortical neurons were plated into 12-well plates at a density of 1.5 × 105 cells/mL. The herbal extracts of Sesami Semen Nigrum (SSN) and Longan Arillus (LA) in a 1 : 1 ratio, i.e., 10 *μ*g/mL SSN + LA referring to 5 *μ*g/mL each, were employed. The combined herbal extracts were added to the cultures in DIV3 for another 3 days of treatment. Immunostainings of synapsin-1 and MAP2 were performed after 4% paraformaldehyde fixation. Images were taken by fluorescent microscopy (upper panel), and the quantification of axonal length and synapsin-1 puncta (low panel) were plotted. Values are expressed as percentage of increase against the untreated control, in mean ± SEM, *n* = 4. (*∗*) *p* < 0.05.

The neurotrophic function of herbal mixtures on neurogenesis was validated in cultured rat primary cortical neurons. Cortical neurons were cultured to enable the progenitor cells to become neurons. Here, the expressions of synapse-1 and MAP2, serving as biomarkers of synaptogenesis and differentiation, respectively, were recognized by fluorescent immunostaining (Figure 4). The treatment of herbal mixture higher than 1 *μ*g/mL significantly promoted the axonal length and the puncta density of synapsin-1 the induction was as good as the scenario of applied NGF at high concentration (Figure 4).

### 3.2. The Herbal Mixture Reverses Depression-like Symptoms in Rat

The SD rats were randomly divided into five groups (*n* = 10). The control group (untreated blank group) and the CMS model group were given saline. For the other three groups, the herbal mixture (Sesami Semen Nigrum + Longan Arillus) at low dose (0.3 g/kg/day), high dose (3 g/kg/day), and fluoxetine (7.2 mg/kg; a positive control) were intragastrically given 30 min before the stress exposure for 6 weeks. Two animal behavior tests, including the sucrose preference test and the forced swimming test, were employed to evaluate the function of herbal extract against depression in the rat. The toxicity of herbal mixtures (low dose: 3 g/kg/day; medium dose: 10 g/kg/day; and high dose: 30 g/kg/day) has been investigated through chronic and acute toxicity tests, and both tests showed no toxicity on the rats (data not shown).

**Figure 5:**
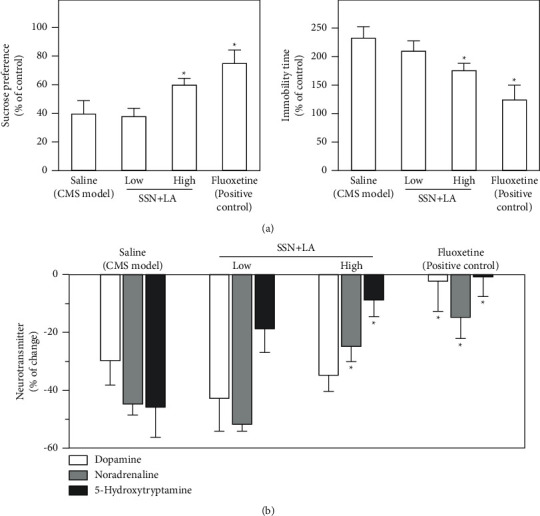
Combined extracts of Sesami Semen Nigrum and Longan Arillus alleviates the depression-like symptoms. The CMS-induced depressive rats were randomly divided into five groups: untreated control, CMS model, herbal mixture at low dose (0.3 g/kg/day), herbal mixture at high dose (3 g/kg/day), and fluoxetine (a positive control at 7.2 mg/kg/day). The preparation of herbal extracts of Sesami Semen Nigrum (SSN) and Longan Arillus (LA) is described as in Figure 3. (a) At the end of CMS procedures, the sucrose preference test (left panel) and forced swimming test (right panel) were carried out, as described in the method session. (b) The amounts of dopamine, norepinephrine, and 5-hydroxytryptamine in the striatum extracts of depressive rats were determined. Values are expressed in percentage of change against the nonstressed control group, in mean ± SEM, *n* = 8. Statistical significance was determined against the CMS-model group. (*∗*) *p* < 0.05.

After the treatment of the herbal mixture, the depression-like symptoms in CMS-induced depressive rats were restored. For the sucrose preference test, the tortured rats showed decreased sugar water consumption as compared with the nonstressed control rats. High dose of herbal extract of Sesami Semen Nigrum + Longan Arillus reversed the sucrose consumption, which was similar to the curative effect of fluoxetine, a positive control (Figure 5(a)). In forced swimming test, the CMS-induced depressive rats doubled cumulative immobility time, while the high dose of herbal mixture administration significantly reduced the cumulative immobility time (Figure 5(a)).

Depression is closely associated with the reduced amount of neurotransmitters in the brain [18]. In the CMS-induced depressive rat brain, the levels of dopamine, noradrenaline, and 5-HT declined by ∼30%, ∼45%, and ∼47%, respectively, which can be effectively restored by the positive control, i.e., intake of fluoxetine (Figure 5(b)). Here, the high dose of herbal extract of Sesami Semen Nigrum + Longan Arillus demonstrated a substantial facilitation of impaired 5-HT level, as well as a significant promotion on the level of noradrenaline (Figure 5(b)). In distinction to fluoxetine, the level of dopamine was not affected by the herbal mixture.

**Figure 6:**
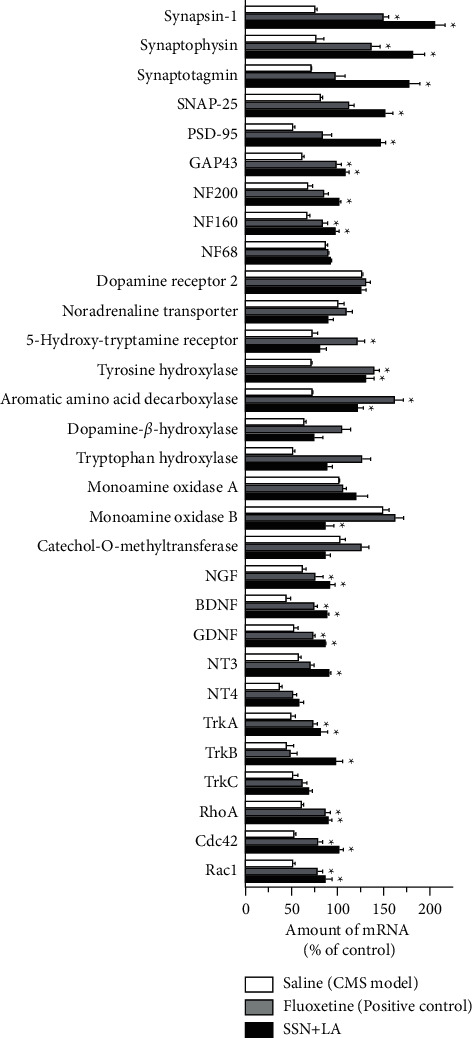
Combined herbal extracts restore the levels of neuronal markers in CMS-induced depressive rat brain. The mRNA levels of neurotrophic factors, neurotrophin receptors, differentiation/synaptogenesis markers, and neurotransmitter receptors/related enzymes in the depressed rat brain were analyzed by real-time quantitative PCR. The preparation of the herbal mixture and the treatment were as in Figure 5. Values are presented in the percentage of the unstressed control group, in mean ± SEM, *n* = 4. Statistical significance was determined against the CMS-model group. (*∗*) *p* < 0.05.

Neurotrophic factors and their corresponding receptors, as well as the abundance of neurotransmitter-related enzymes, play an important role in supporting the maintenance of neuronal functions and which are downregulated in the brain under depression [19, 20]. The depression-related proteins were determined in the brains of herbal-treated rats by RT-PCR with specific primers (Table S1). As shown in Figure 6, high dose of the herbal extract greatly increased the mRNA levels of growth factors, i.e., NGF, BDNF, GDNF, NT3, as well as the high-affinity neurotrophic factor receptors, i.e., TrkA and TrkB. Besides, the high dose of extract also markedly increased the mRNA expressions of differentiation markers, i.e., NF200, NF160, GAP43, and synaptogenesis markers, i.e., synapsin-1, synaptophysin, synaptotagmin, SNAP-25 and PSD-95: the results supported the potential of herbal mixture in promoting neurogenesis *in vivo*. Furthermore, the transcriptional levels of Rho GTPases, i.e., RhoA, Cdc42, and Rac1, were elevated as well, whereas dopamine receptor, noradrenaline transporter, and 5-hydroxytryptamine receptor showed insignificant changes (Figure 6). In many scenarios, the upregulation of these mRNAs could be revealed in the treatment of fluoxetine. Besides, the induction of those neuronal growth proteins in the rat brain was in line with the results in cultured neurons.

## 4. Discussion

Historically, herbal medicines have been widely used in treating shock, low mood, forgetfulness, anhedonia, and dizziness for a long time: these syndromes are similar to mental depression today. In the Chinese medicinal record, the depressive symptom is the first recorded ∼2,500 years ago, where mental depression is considered as an “emotional disease” and caused by insufficiency of “Qi,” i.e., vital energy [21, 22]. In Chinese medicinal history, the herbal prescriptions, such as Xiao-Chai-Hu-Tang, Bai-he-Ji-zi-huang-Tang, and Kai-Xin-San, have been reported to relieve depressive symptoms, and the functionalities of these herbal mixtures have been illustrated in cultured cell and animal models [23–25]. However, the popularization of those products is suffering from two of the aforesaid limitations, i.e., potential toxicity and high price. Here, we successfully come up with a new herbal mixture completely derived from food ingredients, i.e., the combination of extracts from Sesami Semen Nigrum and Longan Arillus, which shows synergy in relieving the symptoms of depression. The sources of Sesami Semen Nigrum and Longan Arillus are easy to get, and the price is much cheaper, as compared to other drugs. Regarding drug registration, these two herbs are considered as food, which therefore can accelerate the registration procedure because of their safety record. Thus, the herbal mixture of Sesami Semen Nigrum and Longan Arillus could be developed as a safe health product to fight against mental depression.

Longan Arillus was reported to enhance memory and alleviate Alzheimer's disease in mice [26, 27]. The main bioactive substances in Longan Arillus are polysaccharides, polyphenols, and flavonoids [28]. In particular, adenosine within Longan Arillus was reported to account for its anxiolytic-like effects [29]. On the other hand, Sesami Semen Nigrum extract, or its oil, has pharmacological evaluation in antihypertension, inhibiting cholesterol absorption/biosynthesis, antioxidation, neuroprotection, and anti-inflammatory [30–32]. Sesamol, sesamin, and sesamolin are the major bioactive compounds in Sesami Semen Nigrum. Among them, sesamol was reported to have significant neuroprotective activity [33] and sesamin was shown to possess the function of stimulating NGF-induced neurogenesis [34]. In *Caenorhabditis elegans*, both sesamin and sesamolin have shown protective ability in alleviating A*β*-induced toxicity and reducing the formation of A*β* oligomers [35]. Thus, the aforementioned evidence is supporting the proposed formulation of combined Sesami Semen Nigrum and Longan Arillus in treating brain diseases.

In cultured PC12 cells, the herbal mixture showed marked stimulation in inducing cell differentiation. We infer that the neurotrophic function of herbal extract could be better when being used in combination with NGF because the results demonstrated the increased expression of neurotrophins and their receptors in the CMS-induced depressive rats upon administration of the herbal mixture. Meanwhile, the extracts of Sesami Semen Nigrum and Longan Arillus showed interaction with NGF, according to our previous reports on HerboChip® drug screening, wherein NGF was employed as a drug target to search for effective herbal extracts [5, 6]. Thus, there may be certain components within the extracts that could bind with NGF and enhance the NGF-induced signaling and function. Besides sesamin, as previously reported [34], none of the single chemicals tested within the herbal mixture exhibited the ability to induce differentiation of PC12 cells alone or even with the help of a low concentration of NGF (data not shown). Thus, the effective substance within the herbal mixture as well as its functioning mechanism have to be further investigated.

## 5. Conclusion

This study provides a new herbal mixture of Sesami Semen Nigrum and Longan Arillus with a weight ratio of 1 : 1. The bioactivities of the herbal mixture have been experimentally validated in cultured PC12 cells, rat cortical neurons, and in CMS-induced depressive rats. The herbal mixture is exhibiting a significant curative efficacy on mental depression with much lower cost and superior safety. The results support the notion of further development of the herbal mixture of Sesami Semen Nigrum and Longan Arillus as therapeutics for depression.

## Data Availability

The data used to support the findings of this study are available from the corresponding author upon request.

## Supplementary Materials

Supplementary Materials Table S1: primers used in real-time PCR analysis.
