# Supplementary material for: A Herbal Mixture of Sesami Semen Nigrum and Longan Arillus Induces Neurite Outgrowth in Cultured Neurons and Shows Anti-Depression in Chronic Mild Stress-Induced Rats
Source: Evid Based Complement Alternat Med. 2022 Jun 16;2022:8809546. doi: 10.1155/2022/8809546 (PMC9225919; doi:10.1155/2022/8809546)

| **Table S1: primers used in real-time PCR analysis.** | |
| --- | --- |
| **Primer** | **Sequence (5’-3’)** |
| GAPDH-S | AAC GGA TTT GGC CGT ATT GG |
| GAPDH-AS | CTT CCC GTT CAG CTC TGG G |
| NGF-S | CAC TCT GAG GTG CAT AGC GTA ATG TC |
| NGF-AS | CTG TGA GTC CTG TTG AAG GAG ATT GTA C |
| BDNF-S | GAG CTG AGC GTG TGT GAC AGT ATT AG |
| BDNF-AS | ATT GGG TAG TTC GGC ATT GCG AGT TC |
| GDNF-S | GCG CTG ACC AGT GAC TCC AAT ATG |
| GDNF-AS | CGC TTC ACA GGA ACC GCT ACA ATA TC |
| NT-3-S | ACA AGC TCT CCA AGC AGA TGG TAG ATG |
| NT-3-AS | TCT CCT CGG TGA CTC TTA TGC TCT G |
| NT-4-S | TCA GTA CTT CTT CGA GAC GCG CTG |
| NT-4-AS | GGC ACA TAG GAC TGT TTA GCC TTG CAT |
| NF68-S | AGC AGA AGA GGA GGA GAA GGA GAA AGA |
| NF68-AS | CTC AAC TGG TTG GTT TGG TGA TGA GGT |
| NF160-S | AGC TGG AAA ATG AGC TTC GGG GAA CA |
| NF160-AS | CTC CAC AAA TTT GTG TTG GAC CTT GAG C |
| NF-200-S | GTG AGT GGA CAT GGA GGG AAT TTT TGG |
| NF-200-AS | GTG AGT GGA CAT GGA GGG AAT TTT TGG |
| GAP-43-S | ACC ACT GAT AAC TCG CCG TC |
| GAP-43-AS | CTA CAG CTT CTT TCT CCT CCT C |
| Synapsin-1-S | CCA GTT CTC CCA GGA CAT TGG A |
| Synapsin-1-AS | GAG GCA TAG TTC CTG AGC TTG TCC |
| SNAP-25-S | GCC AAA GTT CTG GGA TCT CAA GAA G |
| SNAP-25-AS | CAC CAG TGA TCT TGA GCA GAC CA |
| Synaptophysin-S | CTT TCT GGT ACA GCC GTG AG |
| Synaptophysin-AS | ACA GGG TCC CTC AGT TCC TT |
| Synaptotagmin-S | GCT GGG TGA CAT CTG TAC CTC C |
| Synaptotagmin-AS | CAC CTG GAC TTT CTG GAT CTG CTC |
| PSD-95-S | TGG TGA CGA AGA GTG GTG GCA AG |
| PSD-95-AS | CAA AGT GGT AAT CCC GGC CGT C |
| TrkA-S | ACC TCA ACC GTT TCC TCC GGT C |
| TrkA-AS | CTC GAT CGC CTC AGT GTT GGA GA |
| TrkB-S | CGG GAG CAT CTC TCG GTC TAT G |
| TrkB-AS | CAA ATG TGT CCG GCT TGA GCT GG |
| TrkC-S | CAC TGT CTA CTA CCC TCC ACG TG |
| TrkC-AS | CTC TCT GGA AAG GGC TCC TTA AGG |
| Monoamine oxidase A-S | GCC AAA GTT CTG GGA TCT CAA GAA GC |
| Monoamine oxidase A-AS | CAC CAG TGA TCT TGA GCA GAC CAG |
| Monoamine oxidase B-S | GAG AAG AAC TGG TGT GAG GAG CAG |
| Monoamine oxidase B-AS | AGC TGT TGC TGA CAA GAT GGT GGT |
| Tyrosine hydroxylase-S | CCA GTT CTC CCA GGA CAT TGG AC |
| Tyrosine hydroxylase-AS | GAG GCA TAG TTC CTG AGC TTG TCC |
| Dopamine-β-hydroxylase-S | GAA GAA TGC TGT GAC TGT CCA CCA G |
| Dopamine-β-hydroxylase-AS | CAG AGG CTG CAG GTT CCA GTT AC |
| Catechol-O-methyltransferase-S | GGT GAC GCG AAA GGC CAA ATC ATG |
| Catechol-O-methyltransferase-AS | CAG GCC ACA TTT CTC CAG GAG AAG |
| Tryptophan hydroxylase-S | CAC CCA GGA TTC AAG GAC AAC GTC |
| Tryptophan hydroxylase-AS | CAC TGT GAA GCC AGA TCG CTC TTT C |
| Aromatic amino acid decarboxylase-S | GTT GTC ACC CTA GGA ACC ACA TCT TG |
| Aromatic amino acid decarboxylase-AS | CTC ATG AGA CAG CTT CAC GTG CTT TC |
| Dopamine receptor 2-S | AAC TGT ACC CAC CCT GAG GAC ATG |
| Dopamine receptor 2-AS | CTG TCA GGG TTG CTA TGT AGG CC |
| 5-Hydroxytryptamine receptor-S | CAT CAG CAA GGA CCA CGG CTA C |
| 5-Hydroxytryptamine receptor-AS | GGA AGG TGC TCT TTG GAG TTG CC |
| Noradrenaline transporter-S | CAG GTT CAG CAA TGA CAT CCA GCA G |
| Noradrenaline transporter-AS | GTG ATT CCG TAG GCC ACT CTC TC |
| RhoA-S | GGA AGA AAC TGG TGA TTG TTG G |
| RhoA-AS | CTC AAA AAC CTC TCT CAC TCC G |
| Rac1-S | GCA GGC CAT CAA GTG TGT G |
| Rac1-AS | AGG AGG GGG ACA GAG AAC G |
| CDC42-S | TGT TGG TGA TGG TGC TGT TG |
| CDC42-AS | CTT CTT CGG TTC TGG AGG CT |

**graphical abstract**


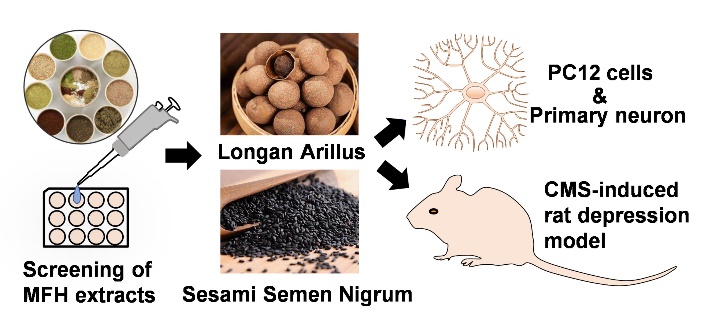

Supplement: Supplementary Materials — Table S1: primers used in real-time PCR analysis. [file 8809546.f1.docx]
